# Menstrual blood loss as an initial trigger for adaptation of iron metabolism in eumenorrheic female athletes—An exploratory study

**DOI:** 10.14814/phy2.70522

**Published:** 2025-08-26

**Authors:** Svenja Nolte, Celina Maier, Simon Klügel, Christopher Weyh, Sebastian Hacker, Claire Badenhorst, Karsten Krüger

**Affiliations:** ^1^ Department of Exercise Physiology and Sports Therapy, Institute of Sport Science Justus‐Liebig‐University Giessen Giessen Germany; ^2^ School of Sport, Exercise and Nutrition Massey University Auckland New Zealand

**Keywords:** erythropoietic response, iron homeostasis, menstrual cycle monitoring, performance

## Abstract

Iron deficiency is a risk factor for impaired performance and recovery. While exercise‐related iron losses are well‐documented, the role of menstrual blood loss (MBL) as a physiological trigger of iron regulation remains underexplored. This study examined whether MBL in eumenorrheic female athletes induces measurable hematological and iron‐related responses, accounting for sex hormone fluctuations. Ten regional‐level football players underwent menstrual cycle tracking and venous blood sampling in both the early follicular and mid‐luteal phases. Hematological parameters, iron markers (ferritin and hepcidin), erythropoietic markers (erythropoietin and reticulocytes), and inflammatory markers (myeloperoxidase [MPO] and C‐reactive protein [CRP]) were measured. MBL was assessed using the Pictorial Blood Loss Assessment Chart (PBAC). Analyses included descriptive statistics, correlation, and linear mixed modeling. MBL was significantly associated with ferritin (*β* = −0.289, *p* = 0.001), reticulocyte counts (*β* = 0.004, *p* = 0.019), and reticulocyte production index (*β* = 0.004, *p* = 0.027). MPO and CRP showed inverse correlations with MBL, suggesting potential immunomodulatory effects. No interaction between MBL and cycle phase was found. MBL appears to stimulate compensatory erythropoiesis in female athletes, largely independent of hormonal phase. Incorporating MBL assessment into athlete monitoring may support individualized strategies to maintain iron balance and optimize performance.

## 1 INTRODUCTION

Despite growing interest in sex‐specific performance optimization, the physiological needs of female athletes remain underrepresented in sports science, particularly regarding factors that directly impact training adaptation and competitive performance. Iron deficiency is a striking example: iron plays a central role in oxygen transport, mitochondrial energy metabolism, and recovery, making it essential for endurance capacity and athletic output. Yet female athletes are significantly more prone to iron depletion and deficiency, with prevalence rates ranging from 15% to 35%, compared to 3% to 11% in males (Sim et al., 2019). These figures highlight a critical and often overlooked lever for performance enhancement: addressing iron deficiency in female athletes represents a clear low hanging fruit. A physiologically meaningful yet readily addressable factor with the potential for substantial impact. Female‐specific factors significantly influence iron status and the risk of deficiency (Sims et al., 2022). Despite this, these physiological and hematological factors remain underexplored in sports science. Targeted research in this area is urgently needed to support both health and performance in female athletes.

It is important to recognize that the hematological system and iron metabolism are under considerable strain in both female and male athletes engaging in exercise or high‐performance sport training. The underlying causes are multifactorial and include increased iron loss through sweat, gastrointestinal micro bleeding, and hemolysis, as well as inadequate dietary intake (Peeling et al., 2008). In elite sports, this problem is particularly pronounced, as high training loads and intense exercise can accelerate iron losses, which are caused by multifactorial mechanisms (Damian et al., 2021). This negative iron balance can be exacerbated by changes to iron regulation in response to the transient acute inflammatory response following exercise. While moderate activity can promote anti‐inflammatory effects, prolonged or intense exercise provokes a pro‐inflammatory milieu, thereby intensifying the problem when the intensity is very high and recovery time is often insufficient. This includes elevated cytokine production, which stimulates hepatic expression of hepcidin, a peptide hormone that serves as the master regulator of iron homeostasis (Ganz, 2003). Hepcidin restricts dietary iron absorption and sequesters iron in storage sites, reducing circulating iron availability in a process termed nutritional immunity. This adaptive mechanism, while protective in the context of infection, may inadvertently limit iron availability for erythropoiesis and metabolic demands in athletes (Ganz & Nemeth, 2024).

Against this background, female athletes face a unique additional burden: menstrual bleeding as the main source of iron loss. Menstrual iron losses are estimated to range from 10 to 40 mg per cycle (Angeli et al., 2016) and can therefore significantly impact iron balance, especially when combined with exercise‐induced losses and increased erythropoietic demands (Bruinvels et al., 2021). Despite its physiological relevance, menstrual status is frequently omitted in iron‐related sports science research—indeed, 65% of studies on iron supplementation fail to report menstrual cycle phase or status (Smith et al., 2022). Preliminary evidence has suggested that iron regulation may vary across the menstrual cycle. Notably, the IronFEMME research group reported phase‐dependent changes in iron status markers but did not observe consistent effects of estrogen on hepcidin levels or post‐exercise iron responses, suggesting a more complex interplay (Alfaro‐Magallanes et al., 2022; Barba‐Moreno et al., 2020). However, it is important to note that participants in these studies generally exhibited low baseline iron status and stores, a factor that may have confounded the interpretation of hormonal influences on hepcidin. Given that iron status itself is considered one of the strongest predictors of hepcidin activity, it is plausible that fluctuations in hepcidin in these cohorts were primarily driven by iron demand, with secondary modulation by other factors such as inflammation, altitude, or sex hormones. In this context, menstrual blood loss, characterized by acute iron loss and increased iron demand, may exert a stronger influence on hepcidin regulation than fluctuations in sex steroid hormones alone.

Therefore, in this study we investigate menstrual blood loss as a potential trigger for acute hematological and iron‐regulatory adaptations. Specifically, we examine whether menstruation in eumenorrheic female athletes induces a compensatory erythropoietic response, potentially mediated by shifts in iron metabolism. A better understanding of these dynamics could help inform individualized strategies for iron monitoring and supplementation in female athletes.

## 2 METHODS

### 2.1 Subjects

Ten female football players (age: 24.5 ± 4.4 years; BMI: 22.8 ± 1.4 kg/m) from two regional football clubs were recruited. All athletes had been playing competitively for several years and were actively competing in the third and fourth tiers of the German women's football league system at the time of data collection. Included participants met the following criteria: (a) healthy adult females between 18 and 35 years; (b) no acute or chronic infection; (c) no consumption of medication; (d) regular menstrual cycles, defined by Elliott‐Sale et al. (2021) as a cycle length of 21–35 days; (e) females with eumenorrhea (no hormonal contraception or implants for at least 6 months prior to initial tracking). To confirm eligibility, participants completed a self‐reported questionnaire on contraception. The study received approval from the local ethics committee of the University of Giessen (No. 2024‐0014) and was conducted following the Declaration of Helsinki for human research. Prior to participation, all individuals were informed about the potential risks and benefits of the study and provided written informed consent, as per institutional guidelines.

### 2.2 Menstrual cycle tracking

**FIGURE 1:**
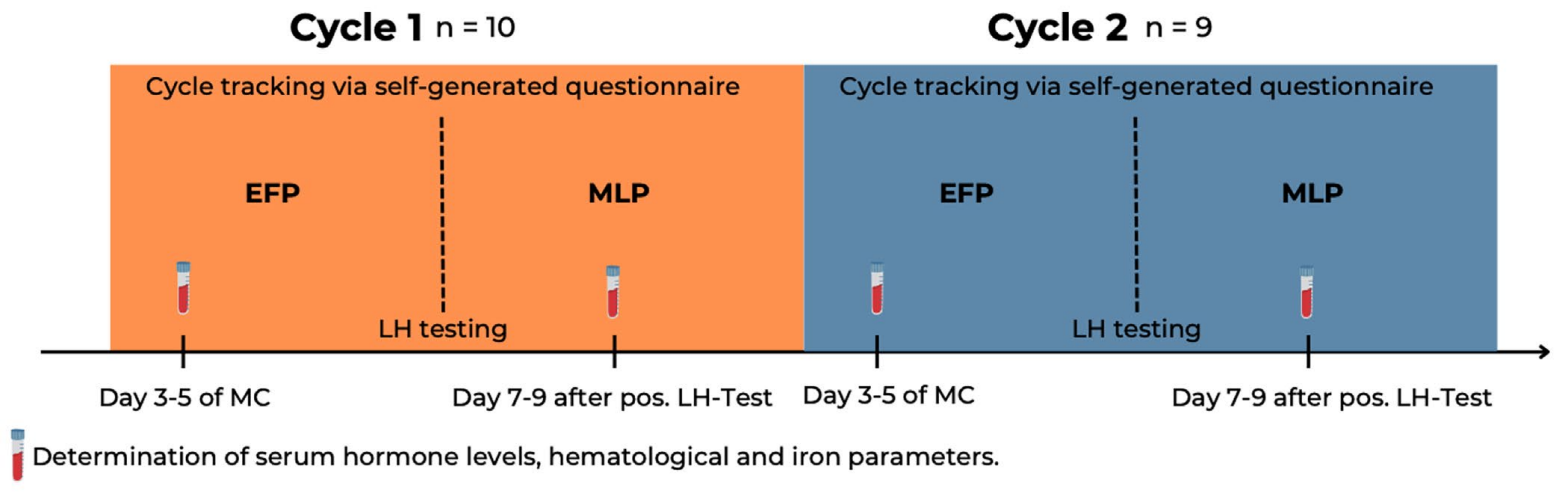
Study design.

Menstrual cycle tracking followed a strict, three‐step protocol described in Peinado et al. (2021). Prior to initiating the tracking process, all athletes completed a questionnaire assessing their use of hormonal contraception or implants, the onset of menarche, history of pregnancy, and the perceived regularity of their menstrual cycles. Five months prior to the scheduled blood sampling, all participants were trained in the symptothermal method for cycle tracking. This method combines daily monitoring of basal body temperature, cervical mucus consistency, and other fertility signs such as cervical position to estimate ovulation and determine fertile and infertile phases of the menstrual cycle. From that point onward, they completed a standardized self‐report questionnaire documenting these fundamental cycle characteristics, which was then manually summarized by the study teams. The collected data were used to determine the optimal timing for luteinizing hormone (LH) testing, which was initiated after 3 months of tracking. Participants received email notifications from the study team with instructions on when to begin and conclude LH testing and were required to submit a photograph of their positive test result. The study team determined whether the LH test result according to the photograph should be considered positive. This combined procedure—symptothermal tracking and LH testing—was conducted over two consecutive cycles. In the third and fourth cycles, participants were scheduled for blood sampling at two time points within the menstrual cycle while continuing to adhere to both tracking steps (symptothermal and LH‐testing) (Figure 1).

### 2.3 Blood sampling and analysis

Participants underwent two blood draws: the first during the early follicular phase (EFP; cycle days 3–5), and the second during the mid‐luteal phase (MLP), scheduled 7–9 days after a positive urinary luteinizing hormone (LH) test. Athletes were classified as eumenorrheic if they exhibited a positive LH test, met the single time point progesterone threshold (≥16 nmol·L^−1^), and had a luteal phase that was longer than 10 days (Elliott‐Sale et al., 2021).

All blood samples were collected between 7:00 and 9:00 a.m. after an overnight fast. Participants were instructed to refrain from intense exercise the day before each sampling and to ensure a minimum of one rest day following their most recent competition. A brief questionnaire was administered to assess recent training load and current health status to rule out subjective feelings of an acute illness or infection. Three venous blood samples were collected in 7.5 mL Serum Gel, two 7.5 mL EDTA, and 2.7 mL EDTA tubes (Sarstedt, Nümbrecht, Germany). The 7.5 mL EDTA samples were immediately centrifuged at 2500*g* for 10 min at room temperature. Plasma was aliquoted into Eppendorf tubes, placed on dry ice for transport, and subsequently stored at −80°C until analysis.

Hormonal parameters—including testosterone (measured once in EFP), follicle‐stimulating hormone (FSH), luteinizing hormone (LH), estradiol, and progesterone‐were analyzed via chemiluminescent immunoassay (CLIA) from plasma samples by an accredited medical laboratory (SYNLAB Medical Care Center, Bad Nauheim, Germany).

Hemoglobin, hematocrit, and red blood cell count were measured using automated impedance and photometric methods (ADVIA systems). Ferritin, transferrin, and soluble transferrin receptor (sTfR) were quantified using immunoassays (ELISA); the ferritin index (sTfR/log ferritin) was calculated accordingly, and hepcidin was analyzed by liquid chromatography coupled with mass spectrometry (LC–MS). In addition, markers of neutrophil activation (myeloperoxidase (MPO) and lactoferrin) were quantified from 50‐μL plasma samples in duplicates using a magnetic Luminex ELISA assay (Bio‐Techne Ltd., Cat# LXSAHM‐08, Abingdon, Oxon, UK).

### 2.4 Menstrual blood loss validation

To validate the assessment of menstrual blood loss (MBL), a pictorial blood loss assessment chart (PBAC) was used. The PBAC serves as a noninvasive and cost‐effective tool for estimating MBL by recording the usage and degree of saturation of single‐use sanitary products. Based on the PBAC entries, the Higham Score was calculated, which serves as an objective and standardized measure of MBL (Higham et al., 1990; Zakherah et al., 2011).

Participants were provided with the same brand of single‐use sanitary products to minimize variability in absorbency and instructed to document their menstrual bleeding using the PBAC over the course of the two cycles where venous blood samples were collected. As part of the PBAC, participants were asked to report the number of tampons and pads used, along with their degree of saturation (light, moderate, or fully soaked). Additionally, they were asked to document the occurrence and frequency of menstrual clot passage. The completed PBAC was returned to the study team at the time of the blood draw during the EFP.

### 2.5 Statistical analysis

All statistical analyses were conducted using Microsoft Excel (Microsoft Corporation, Redmond, WA, USA) and JASP (Version 0.17.2.1, University of Amsterdam, Netherlands). Data are presented as mean ± standard deviation (SD), unless otherwise stated. Normality of distribution was assessed using the Shapiro–Wilk test. To evaluate the consistency of repeated measurements across two consecutive menstrual cycles, intraclass correlation coefficients (ICCs) were calculated. The results indicated moderate‐to‐high agreement for most variables, supporting the use of both cycles in the statistical models. This approach ensured the inclusion of biologically plausible variation while improving statistical reliability and power.

To examine differences between the early follicular phase (EFP) and the mid‐luteal phase (MLP), paired *t*‐tests were applied for normally distributed variables; otherwise, the nonparametric Wilcoxon signed‐rank test was used.

A quotient of serum ferritin (sFer) to MBL was calculated to explore the relationship between iron stores and individual bleeding volume. This index was developed to facilitate a more practical interpretation of iron status in athletes.

To assess associations between variables, Pearson correlation coefficients were calculated for normally distributed data. These correlations were used to explore relationships between menstrual blood loss (MBL), reticulocyte (%), quotient (sFer/MBL), and EPO and hepcidin.

All tests were two‐tailed, and statistical significance was defined as *p* < 0.05.

Figures and visualizations were generated using R (Version 4.5.0, R Foundation for Statistical Computing, Vienna, Austria) and JASP.

In a final exploratory step, potential associations between variables of iron metabolism, hematological parameters, cytokines, hormones, and menstrual blood loss (MBL) were analyzed using linear mixed models (LMM). The models of LMM accounted for both fixed and random effects, allowing for the dependency of repeated measurements within participants to be appropriately modeled. The dependent variables were grouped into four categories: iron metabolism parameters, hematological parameters, cytokines, and hormones. Fixed effects included menstrual cycle phase and menstrual blood loss (MBL). Additionally, an interaction term between cycle phase and MBL was included to test whether the impact of cycle phase on the dependent variables varied according to the extent of menstrual blood loss. This interaction was specified based on the hypothesis that greater blood loss during menstruation might influence iron status, as well as hematological and immunological parameters, in a cycle‐phase‐dependent manner. To control for interindividual differences, a random intercept was included in the model. Maximum likelihood estimation (ML) was used, and likelihood ratio tests assessed the significance of model terms. Unstandardized regression coefficients (B) were reported for the fixed effects, reflecting the practical relevance of findings in the original measurement units of the dependent variables. The significance level was set at *α* = 0.05.

## 3 RESULTS

### 3.1 Hormonal and cycle characteristics

Table 1 displays menstrual cycle characteristics, including the cycle length, luteal phase length, and the Higham score for all participants collected over 2 months. Estrogen, progesterone, LH, and FSH concentrations in both the EFP and MLP, measured over two cycles, are summarized in Table 2. Testosterone was measured in the EFP of the first cycle. A significant time effect for estrogen, progesterone, and FSH (*p* < 0.001) between EFP and MLP was found. LH showed no significant time effect across the two cycles.

**TABLE 1:** Menstrual cycle characteristics across two cycles.

Descriptive	Cycle length (days)	Luteal phase length (days)	Higham‐score
Mean (Median)	29.37	13.89	(97.5)
SD (IQR)	4.73	3.56	(57.25)

**TABLE 2:** Hormonal profiles of two cycle phases across two cycles.

Cycle phase	Descriptive	Testosterone (ng/mL)	Estrogen (pg/mL)	Progesterone (ng/mL)	LH (mU/mL)	FSH (mU/mL)
EFP	Mean	0.45	36.89	0.41	4.71	7.11
	SD	0.10	16.84	0.19	1.12	1.33
MLP	Mean		127.28	13.56	4.84	3.51
	SD		49.48	5.92	2.30	1.49

### 3.2 Changes of hematological and iron metabolism parameters in different cycle phases

**FIGURE 2:**
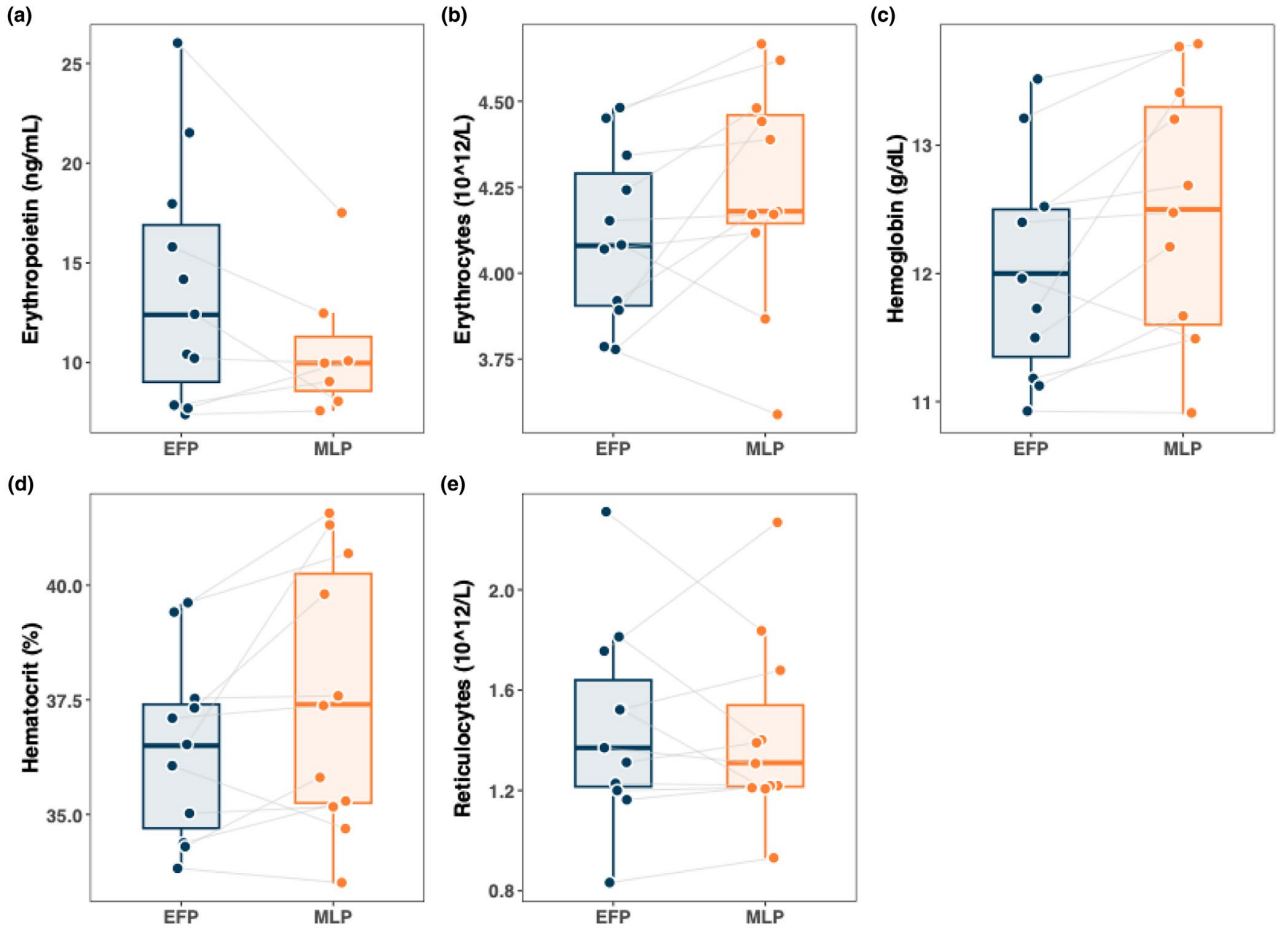
Comparison of hematological parameters between EFP and MLP.

**FIGURE 3:**
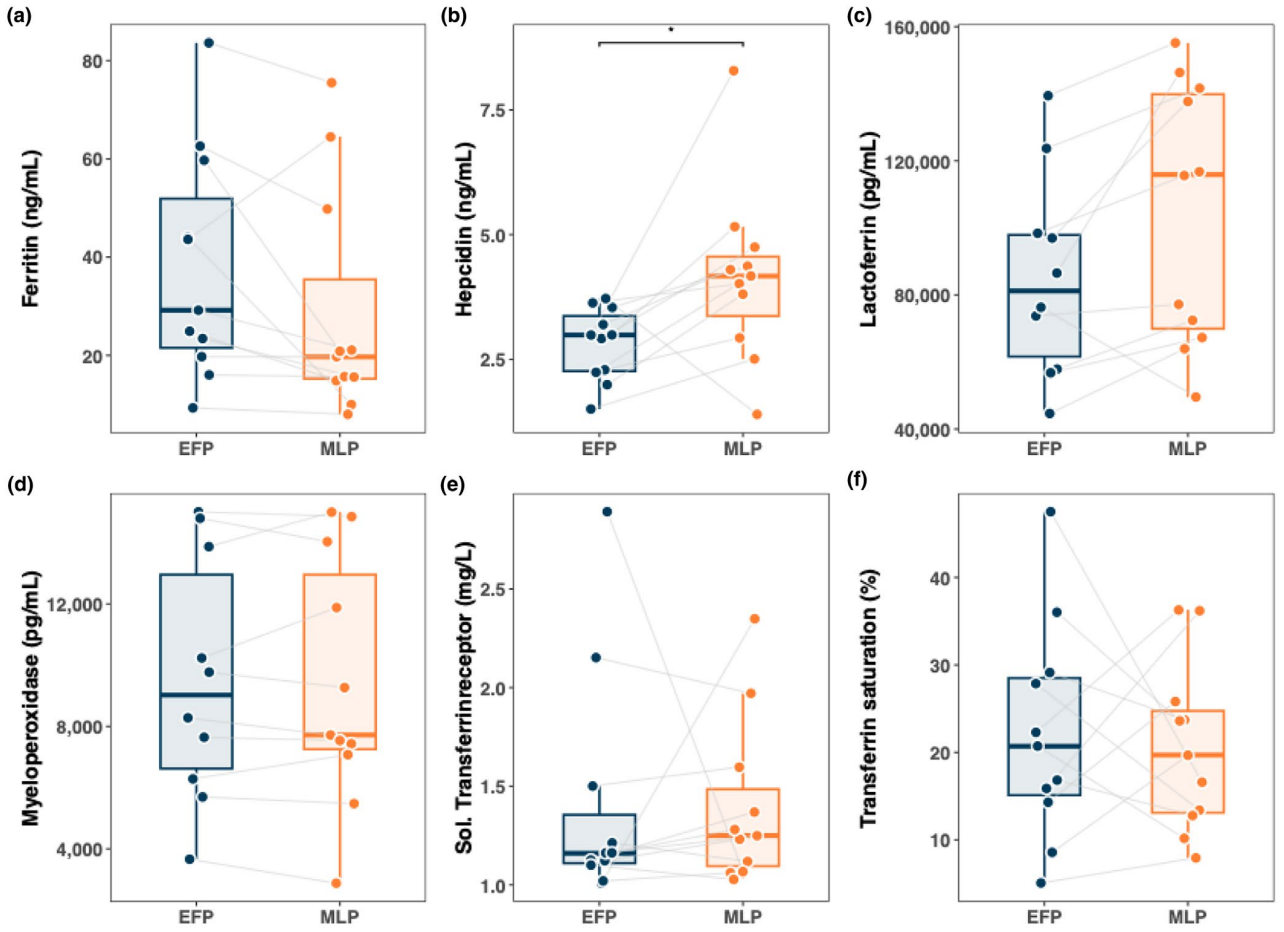
Comparison of iron metabolism parameters between EFP and MLP. Asterisks indicate statistically significant differences (*p* < 0.05).

Figures 2 and 3 illustrate differences in hematological and iron metabolism parameters between the EFP and MLP. Erythropoietin and reticulocytes were higher in the EFP compared to the MLP, whereas erythrocytes, hemoglobin, and hematocrit were higher in the MLP compared to the EFP. However, none of these differences were statistically significant. For ferritin and MPO, nonsignificantly higher values were found in the EFP compared to MLP. Hepcidin, lactoferrin, sTfR, and transferrin were higher in the MLP compared to the EFP without reaching statistical significance. Only hepcidin showed a significant time effect (*p* < 0.05).

**FIGURE 4:**
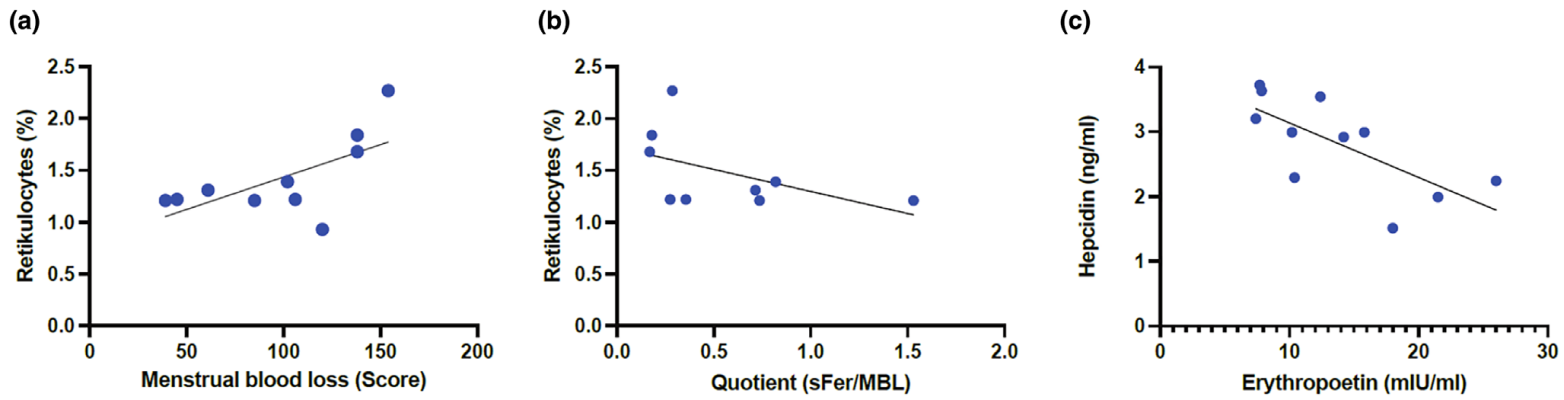
Correlations between hematological variables, menstrual blood loss, and iron metabolism variables.

A significant correlation was found between reticulocytes and menstrual blood loss (Higham Score) (*r* = 0.64, *p* < 0.05), as demonstrated in Figure 4a. In contrast, a nonsignificant association was observed between the percentage of reticulocytes and the quotient sFer/MBL (*r* = −0.61, *p* = 0.09) (Figure 4b). While no correlation was found between menstrual blood loss MBL and EPO, a significant negative correlation was identified between hepcidin and EPO in the EFP (*r* = −0.71, *p* < 0.05) (Figure 4c).

### 3.3 Linear mixed model

**FIGURE 5:**
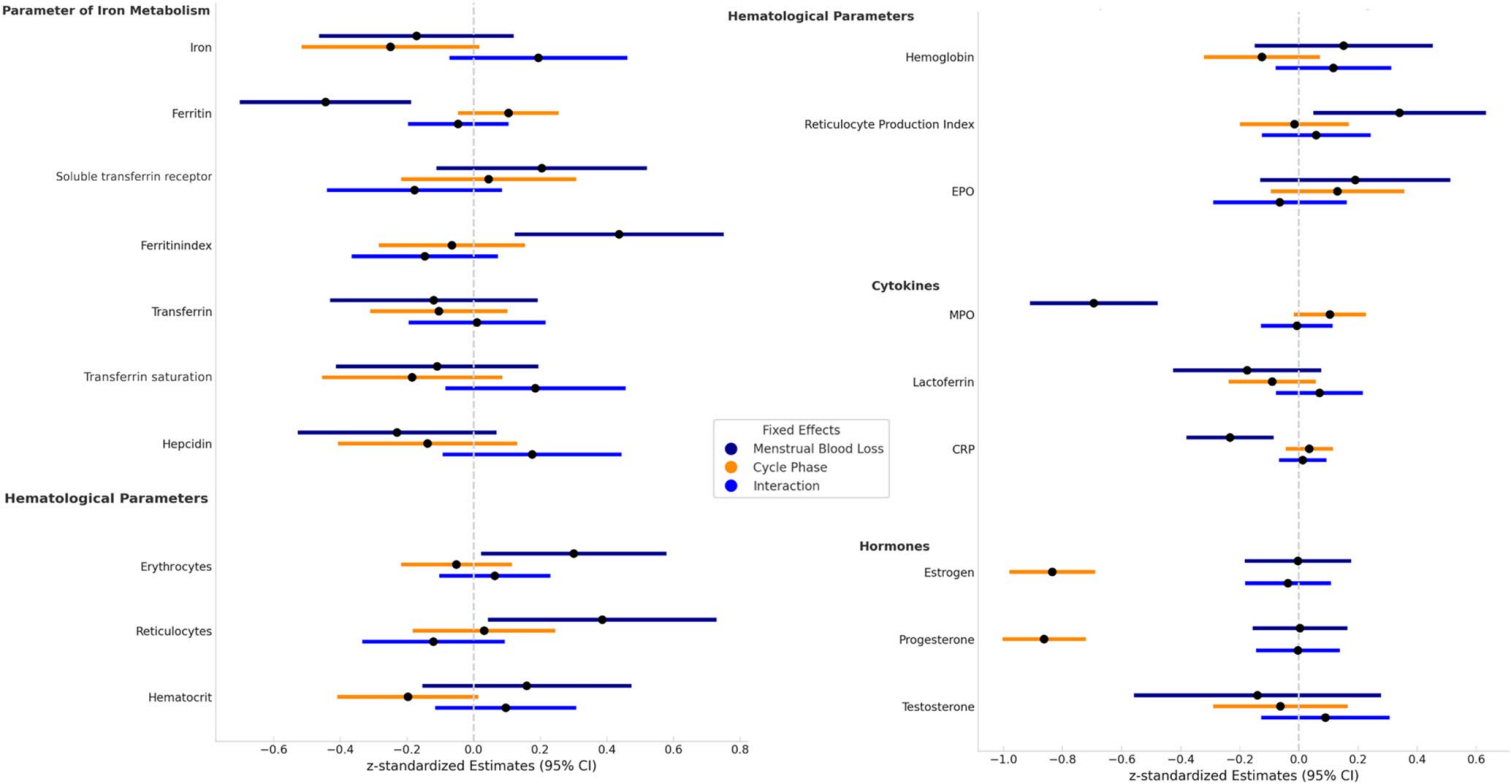
Forest plot illustrating the results of the linear mixed model analyses examining the associations between menstrual blood loss (MBL), menstrual cycle phase, and their interaction with parameters of iron metabolism, hematological markers, cytokines, and hormones. Displayed are standardized regression coefficients (β) and their 95% confidence intervals (CI) for each predictor across outcome variables.

Associations between variables of iron metabolism, hematological parameters, cytokines, hormones, and MBL are demonstrated in Figure 5. Ferritin and the ferritin index showed significant associations with MBL. Specifically, there was a significant negative association between MBL and ferritin levels (*β* = −0.289, SE = 0.085, 95% CI [−0.460, −0.118], *p* = 0.001), indicating that higher menstrual blood loss was linked to lower ferritin concentrations. In contrast, no significant effects of cycle phase or the interaction between cycle phase and MBL were observed for ferritin. Similarly, there was a significant positive association between MBL and the ferritin index (*β* = 0.005, SE = 0.002, 95% CI [0.001, 0.009], *p* = 0.010), suggesting that increased menstrual blood loss corresponded to higher ferritin index values. Again, neither cycle phase nor its interaction with MBL showed significant effects on this parameter. All other analyzed markers of iron metabolism did not reveal any significant associations.

Regarding hematological parameters, we found a significant positive association between MBL and erythrocyte levels (*β* = 0.002, SE = 0.001, 95% CI [0.0001, 0.004], *p* = 0.040), and between MBL and reticulocyte levels (*β* = 0.004, SE = 0.002, 95% CI [0.001, 0.008], *p* = 0.019). Furthermore, a significant positive association was found between MBL and the reticulocyte production index (*β* = 0.004, SE = 0.002, 95% CI [0.001, 0.007], *p* = 0.027). For all three hematological parameters, neither cycle phase nor the interaction between cycle phase and MBL demonstrated significant effects. No further associations were detected in the remaining hematological markers.

Cytokine analysis revealed that higher MBL was significantly and negatively associated with MPO levels (*β* = −69.30, SE = 10.98, 95% CI [−91.55, −47.05], *p* < 0.001), indicating that greater blood loss corresponded to lower MPO concentrations. Neither cycle phase nor its interaction with MBL had a significant effect on MPO. Similarly, MBL showed a significant negative association with CRP levels (*β* = −0.001, SE = 0.000439, 95% CI [−0.002, −0.0003], *p* = 0.003). No significant effects were found for cycle phase or its interaction with MBL in relation to CRP.

For hormonal parameters, estradiol and progesterone were significantly influenced by cycle phase. Estradiol levels were significantly lower in the early follicular phase (EFP) compared to the mid‐luteal phase (MLP) (*β* = −46.69, SE = 12.78, 95% CI [−72.53, −20.84], *p* < 0.001). However, no significant associations were observed between estradiol levels and MBL, nor was there a significant interaction between cycle phase and MBL. Similarly, cycle phase was a significant predictor of progesterone levels (*β* = −6.43, SE = 1.48, 95% CI [−9.44, −3.41], *p* < 0.001), with lower progesterone concentrations found in the EFP compared to the MLP. Again, no significant associations were observed between progesterone levels and MBL, nor was there a significant interaction effect.

## 4 DISCUSSION

Our findings demonstrate that MBL is significantly associated with key hematological and iron‐related parameters. Specifically, greater MBL correlated with lower ferritin concentrations, elevated reticulocyte counts, and higher reticulocyte production index. Such results would suggest a compensatory erythropoietic response to iron loss during menstruation. Moreover, a negative association between MBL and inflammatory markers such as MPO and CRP was found, indicating potential immunological adjustments alongside hematological changes that occur during menstruation.

These findings align with prior evidence that iron metabolism in female athletes is shaped by both training‐related demands and menstrual blood loss (Badenhorst et al., 2021; Pedlar et al., 2018). The novel contribution of this study lies in the observation that even within athletes, variation in MBL can modulate iron availability and erythropoietic activity, independent of cycle phase. This finding aligns with growing research indicating that the magnitude of iron regulation, particularly hepcidin activity, is primarily influenced by iron status and iron demand. Regulating factors, such as sex hormone levels, exert a secondary effect (Sim et al., 2019). Importantly, the lack of interaction effects between cycle phase and MBL suggests that iron‐related adaptations are more directly linked to the magnitude of blood loss than to fluctuating sex hormone levels per se.

### 4.1 Menstrual cycle characteristics and hormonal fluctuations

The mean cycle length, luteal phase duration, and Higham Score fall within established reference ranges (Skiba et al., 2019), suggesting our participants had regular menstrual cycles and typical menstrual bleeding patterns.

Estrogen, progesterone, and FSH showed significant differences between the early follicular phase (EFP) and the mid‐luteal phase (MLP), which reflects the characteristic hormonal fluctuations of the menstrual cycle, low ovarian hormone levels during the EFP and elevated estrogen alongside peak progesterone during the MLP (Guo et al., 2015; Rishpon‐Meyerstein et al., 1968). These hormonal fluctuations must be considered when interpreting downstream physiological processes such as erythropoiesis and iron regulation. In this study, the cohort displayed eumenorrheic sex steroid hormonal concentrations. Previous research has reported variations in iron status throughout the eumenorrheic cycle, with suggested contributions from sex steroid hormone concentrations (Alfaro‐Magallanes et al., 2022). For instance, some studies have indicated that fluctuations in estrogen and progesterone levels may influence iron metabolism and erythropoietic activity (Hamad et al., 2020; Li et al., 2016). However, the associations described in this study appear to be largely independent of the menstrual cycle phase. Despite substantial variations in circulating estrogen and progesterone levels across the cycle, the relationship between cycle phase and iron metabolism was not observed in this data. This suggests that the regulatory mechanisms governing erythropoiesis and iron mobilization in response to menstrual blood loss may operate independently of cyclical hormonal changes. Our results differ from previous findings in that they highlight the primary role of blood loss in modulating iron availability and erythropoietic activity, rather than the secondary effects of sex hormone fluctuations. These findings are consistent with previous research demonstrating that menstrual phase‐related hormone fluctuations did not alter erythropoietic responses in women living at high altitude (Reeves et al., 2001). Nevertheless, it is important to acknowledge that physiological systems are highly interconnected, and subtle or indirect interactions cannot be entirely ruled out.

### 4.2 Hematological and iron regulatory adaptation to menstrual blood loss

Despite cyclical hormonal fluctuations and menstrual blood loss, hemoglobin, hematocrit, and red blood cell (RBC) counts remained stable across the menstrual cycle, indicating that oxygen transport capacity is maintained. This suggests that, in athletes with sufficient iron reserves, menstrual blood loss does not immediately impair hematological parameters.

However, mean ferritin (37.83 ± 23.08 ng/mL) and hemoglobin levels (12.05 ± 0.86 g/dL) measured in the EFP were close to thresholds indicative of suboptimal iron status (Nolte et al., 2024). While these values were sufficient to maintain stable hematological parameters, they suggest a generally lower iron reserve in this athletic cohort, potentially increasing their relative susceptibility to iron deficiency. This is consistent with prior findings showing a higher prevalence of iron deficiency in the female athletic population (Pengelly et al., 2025).

A positive correlation between MBL and reticulocyte count was observed, particularly in the MLP, indicating a compensatory increase in red blood cell production over time. This aligns with the findings from Mullen et al., who showed that reticulocyte percentage was significantly lower in the follicular and ovulatory phase (Mullen et al., 2020). Since erythropoiesis is a time‐dependent process, this temporal relationship supports the physiological mechanism of delayed hematological response to blood loss. The association was further influenced by iron availability: when the ratio of ferritin to MBL was low—suggesting depleted iron stores relative to blood loss—reticulocyte counts were higher. This pattern reflects an increased erythropoietic drive in response to iron stress and emphasizes that iron‐deficient individuals may require a stronger compensatory response to maintain hematological balance. Such a response is characteristic of stress erythropoiesis, a form of emergency red blood cell production triggered by acute or chronic blood loss, iron deficiency, or other physiological stressors (Ruan & Paulson, 2023). In contrast to steady‐state erythropoiesis, stress erythropoiesis is associated with increased EPO activity and enhanced proliferation of erythroid progenitors, often under conditions of limited iron availability (Paulson et al., 2011, 2020). This mechanism appears to be relevant in female athletes, whose physiological baseline includes recurrent blood loss through menstruation combined with athletic training loads. Given the predictable and cyclical nature of menstrual bleeding, it is conceivable—albeit speculative—that such erythropoietic flexibility may reflect an evolutionary adaptation to recurring hematological stress in women. While direct evidence for this hypothesis is currently lacking, the regular activation of erythropoietic mechanisms in response to menstrual blood loss could represent a biologically conserved strategy to preserve oxygen‐carrying capacity and physiological resilience under conditions of repeated iron loss.

Changes in hematological and iron metabolism markers across the menstrual cycle were relatively subtle, with hepcidin being the only parameter to show a significant time effect, higher in the MLP. Beyond temporal effects, several associations emerged independently of the cycle phase. Reticulocytes, erythrocyte numbers, and the reticulocyte production index all showed significant positive associations with MBL. MBL was also positively associated with red blood cell counts, while ferritin and ferritin index were negatively associated, regardless of cycle phase, indicating that the physiological response to MBL operates independently of fluctuating sex hormones. These findings support the concept that menstrual blood loss acts as a key driver of adaptive hematological responses. Iron status and demand thus appear to be the primary regulators of iron metabolism, overriding cyclical hormonal influences. This is consistent with the well‐established notion that iron deficiency is primarily caused by acute or chronic blood loss, with menstruating women representing a high‐risk group (Charlton & Bothwell, 1982; Hallberg & Rossander‐Hultén, 1991). The observed negative association between MBL and ferritin highlights the depletion of iron reserves during menstruation, while a concurrent increase in the ferritin index suggests an attempt to preserve erythropoiesis under iron‐limited conditions (Infusino et al., 2012). Supporting this hypothesis, soluble transferrin receptor (sTfR) concentrations may also rise, reflecting increased cellular demand for iron to support red blood cell production (Harms & Kaiser, 2015). In summary, the combined evidence of stable hemoglobin alongside rising reticulocyte counts, declining ferritin levels, and phase‐independent associations with MBL supports a coordinated erythropoietic adaptation to menstrual blood loss in female athletes. These adjustments are consistent with physiological compensation mechanisms and point to the importance of iron availability in sustaining erythropoiesis during recurring blood loss (Silvestri & Nai, 2021).

### 4.3 Regulation of hepcidin and erythropoiesis

Focusing specifically on the hormonal regulation of iron metabolism, our data revealed a distinct phase‐dependent pattern in the interplay between EPO and hepcidin. In the EFP, when menstrual blood loss likely triggers compensatory erythropoiesis, we observed a significant negative correlation between EPO and hepcidin levels. This aligns well with the physiological model in which EPO stimulates erythropoiesis and concurrently suppresses hepcidin to facilitate iron mobilization, a key mechanism in stress erythropoiesis (Paulson et al., 2020; Ruan & Paulson, 2023).

Interestingly, this regulatory relationship appeared to shift in the MLP. While EPO levels declined, hepcidin concentrations were significantly higher compared to the EFP. This pattern suggests that the erythropoietic drive, and thus the suppressive effect of EPO on hepcidin, diminishes as the cycle progresses. The rise in hepcidin during the MLP may therefore reflect a return to baseline regulation once the acute need for iron mobilization subsides.

One possible contributing factor is the concurrent increase in progesterone levels during the luteal phase, which has been shown to upregulate hepatic hepcidin expression (Li et al., 2016). However, our data do not support a direct association between hepcidin levels and menstrual cycle hormones; suggesting that the observed regulation may occur independently of progesterone fluctuations.

Taken together, these findings support the hypothesis that female athletes may undergo cyclical, low‐grade states of stress erythropoiesis in response to menstrual blood loss, requiring finely tuned hormonal (EPO) and iron regulatory mechanisms to maintain oxygen transport capacity. The elevated hepcidin levels in the MLP, while initially counterintuitive and in contrast to previous literature reporting suppressed hepcidin under erythropoietic stress (Yang et al., 2012), can thus be understood as part of a temporally dynamic regulatory system, influenced by erythropoietic signals.

### 4.4 Inflammatory regulation and immune modulation

Additionally, we found a negative association between myeloperoxidase (MPO), a marker of neutrophil activity, and MBL. Importantly, blood samples were obtained at rest to avoid exercise‐induced transient changes in MPO levels, which have been previously described in the literature (Rooney et al., 2018). By controlling for this factor, we aimed to investigate whether interindividual differences in resting inflammation relate to MBL, rather than short‐term exercise effects. This finding may reflect an iron‐dependent impairment of neutrophil function, as sufficient iron availability is crucial for optimal immune cell activity (Kuźmicka et al., 2022; Maneva & Taleva, 2009).

Furthermore, C‐reactive protein (CRP), a marker of systemic inflammation, has previously been reported to fluctuate across the menstrual cycle, with inverse associations to estrogen and positive associations with progesterone (Wander et al., 2008). In our study, however, CRP was negatively associated with MBL but showed no significant variation according to menstrual cycle phase. It is speculated that the reason for this inverse relationship is that a reduction in CRP—and thus in systemic inflammation—may represent a regulatory strategy to suppress hepcidin levels. Since hepcidin inhibits iron absorption and release, its downregulation would facilitate iron availability for erythropoiesis in the context of increased iron demand (Ganz, 2003), here in response to MBL.

Together, these associations point to a possible role of resting inflammatory tone in iron regulation, independent of acute inflammatory events in menstruating female athletes.

### 4.5 Limitations

While these findings are promising, several limitations should be acknowledged. The relatively small sample size, primarily resulting from logistical challenges and strict methodological criteria, particularly regarding menstrual cycle classification, may limit statistical power and generalizability. Additionally, given the exploratory nature of the linear mixed model, no correction for multiple testing was applied, increasing the potential risk of type I errors. As such, results should be interpreted with appropriate caution and regarded as hypothesis‐generating. Moreover, the current findings are limited to a specific subgroup, eumenorrheic, hormonally non‐contracepting athletes, and may not be directly transferable to nonathletic populations, individuals with menstrual irregularities, or those using hormonal contraception. Nonetheless, the application of z‐standardization across outcomes and visualization of standardized effect sizes in forest plots enhances interpretability and provides a strong foundation for future confirmatory research.

## 5 CONCLUSION

This study supports the notion that iron status and demand are the primary modulators of iron metabolism. In females, MBL may be considered a primary relevant modulator of iron status and demand as higher levels of MBL will negatively affect iron status and increase iron demand; as a result, influence iron metabolism. By quantifying MBL in this population for the first time, we demonstrated that greater blood loss is associated with lower ferritin levels and increased hematological signs of stimulated erythropoiesis, such as elevated reticulocyte and erythrocyte counts. These findings suggest that erythropoietic adaptation to blood loss occurs independently of cyclical sex hormone fluctuations, underscoring MBL as a direct contributor to hematological regulation. Importantly, this study highlights the value of integrating MBL quantification into female athlete health monitoring. Conventional markers like ferritin and hemoglobin may not sufficiently capture ongoing physiological demands or early stages of iron depletion, particularly in the absence of anemia or hypoxia. In contrast, a combined evaluation of MBL, iron storage indicators, and markers of erythropoietic activity such as reticulocyte counts offers a more individualized and physiologically sensitive approach for female athletes.

The findings of this study underscore the value of incorporating MBL as a physiological parameter in athlete health and performance monitoring. Acknowledging individual variability in MBL enables more timely and tailored interventions, ranging from nutritional strategies to optimize iron intake and absorption to temporary modifications in training load during periods of increased physiological strain. Notably, while MBL is a key determinant of iron status, it remains a subjective experience, often misjudged by athletes. The use of standardized tools such as the PBAC can enhance objectivity and facilitate a more accurate assessment in both clinical and applied sports settings.

Rather than being viewed solely as a clinical symptom, MBL should be recognized as a relevant physiological stimulus with direct implications for erythropoiesis and iron metabolism. Future research and athlete care models should integrate MBL assessment into personalized monitoring strategies to safeguard iron homeostasis, support recovery, and optimize long‐term performance in female athletes.

## AUTHOR CONTRIBUTIONS

K.K. was involved in supervision. S.N. and C.M. were involved in conceptualization. S.N., S.K., and C.M. were involved in data collection, data analysis, and data curation. S.N. and C.W. were involved in statistical analysis. S.N., S.H., and C.W. were involved in visualization. S.N. was involved in original—draft preparation. S.N., K.K., C.B., and C.M. were involved in writing—review and editing. All authors approved the final version of the manuscript.

## FUNDING INFORMATION

The study did not receive any external funding.

## CONFLICT OF INTEREST STATEMENT

There is no conflict of interest.

## ETHICS STATEMENT

The study was approved by the local ethics committee of the University of Giessen (No. 2024‐0014).

## INFORMED CONSENT

All subjects provided written informed consent to participate in the study.

## Data Availability

All data are available upon request.
